# Growth standard charts for monitoring bodyweight in intact domestic shorthair kittens from the USA

**DOI:** 10.1371/journal.pone.0277531

**Published:** 2022-11-21

**Authors:** Carina Salt, Alexander J. German, Kristin S. Henzel, Richard F. Butterwick

**Affiliations:** 1 Waltham Petcare Science Institute, Mars Petcare, Waltham on the Wolds, Melton Mowbray, Leicestershire, United Kingdom; 2 Institute of Life Course and Medical Sciences, University of Liverpool, Neston, Cheshire, United Kingdom; 3 Royal Canin Research Center, Aimargues, France; Cairo University Faculty of Veterinary Medicine, EGYPT

## Abstract

The optimal growth of domesticated cats has not yet been well defined. This study first aimed to create evidence-based growth standards for healthy pet domestic shorthair (DSH) kittens, and then compare the pattern of growth curves depicted by the standards with growth patterns in other healthy DSH kittens and those with abnormal body condition. Data were derived from the clinical records of the BANFIELD® Pet Hospital (BANFIELD) network in the USA and from a research population in the UK (Waltham Petcare Science Institute, UK). A ‘modelling’ dataset was first created from the BANFIELD records, comprising bodyweight data from immature client-owned DSH cats that had remained healthy and in ideal body condition within the first 2.5y of life. This was used to construct growth centile curves for male and female kittens, covering the age range 8 to 78 weeks, using Generalised Additive Models for Location, Shape and Scale. Growth patterns depicted by the centile curves were compared with the growth patterns of healthy DSH kittens from both research colonies and kittens attending BANFIELD that were classified as overweight or underweight. Overall, there was a broad agreement to the growth standards with approximately half of the research population (206/507, 49%) staying within 2 centile lines of their starting centile, and upward and downward movements outside this range being roughly equally distributed. Compared with the growth standards, the 122 overweight BANFIELD kittens were heavier on average at the start of monitoring and subsequently grew more quickly with 63 (52%) crossing at least 2 standard centile lines upwards. Only 4 underweight DSH kittens were available in the BANFIELD database; compared with the standards, there was a marked initial dip in growth followed by subsequent catch-up growth and 2/4 kittens crossed 2 or more centile lines downwards at some point. Evidence-based growth standards are developed here for male and female sexually-intact DSH kittens. Crossing centiles in an upwards and downwards direction is associated with cats becoming overweight or underweight by early adulthood, respectively. Further work is required to determine whether the clinical use of these growth standards will improve the health and wellbeing of pet cats.

## Introduction

The growth phase is critical for health in many species including companion animals such as dogs and cats. In humans, slower than expected growth can suggest under-nutrition [1], whilst overly rapid growth and ‘catch-up’ growth are known risk factors for obesity [2–4]. Similarly, malnutrition in puppies and kittens can cause growth retardation [5, 6], whilst over-nutrition can lead to puppies and kittens becoming overweight [5, 7], which is a growing concern in the veterinary profession [8, 9]. However, there is limited information and little current guidance available on what constitutes optimal growth in kittens.

In humans, growth standards, such as those of the World Health Organization (WHO) [8], can be used as a guide for how children are growing and developing. Such standards can also be used to describe the requirements for health and wellbeing of populations, as well as determining and monitoring the effectiveness of interventions or environmental factors [10–12]. Therefore, these are a valuable component of the human paediatric toolkit allowing trained health professionals to compare an individual’s growth and development with that of a healthy reference population [10–12]. Individuals with potential growth disorders can be identified thereby enabling investigations and corrective measures to be undertaken [10–12]; children at risk of developing obesity can also be identified [3, 4]. In recent work, evidence-based growth standards have been developed for male and female dogs of 5 different size categories [5, 13], and the growth curves depicted by these standards have been compared with the growth patterns of dogs that were healthy, had abnormal body condition, or had various diseases with the potential to affect growth [5]. Taken together, the results of this work demonstrate the potential for growth standards as a useful tool for monitoring healthy growth.

To date, there has been little research into growth monitoring in kittens. In previous research, bodyweight was regularly assessed from 3 months to 8.5y in a colony of research cats, and variables associated with overweight status during adulthood were examined [7]. Rate of weight gain between 3 months and 12 months was found to be significantly associated with overweight status in adulthood [7]. In a further study, growth was assessed using non-linear mixed modelling to determine the risk of developing obesity in a second research colony, where cats were housed in sheltered, predominantly outdoor conditions, where they were exposed to seasonal changes in temperature and light [14]. Being born during the increasing photoperiod, being of male sex and bodyweight at 15 weeks were all significantly associated with being overweight at 9 years, but maternal factors, birth weight and litter size were not [14]. Despite this previous research, there remains limited guidance available to veterinary professionals on what constitutes optimal growth in kittens, and growth standards similar to those used for children [10–12] and puppies [5, 13] have not yet been created. In light of this, the first aim of this study was to utilise bodyweight data from a large population of healthy entire pet domestic shorthair (DSH) cats attending veterinary hospitals in the USA to create evidence-based growth standards for kittens. The second aim was to compare growth curves depicted by these standards with growth patterns in a separate population of healthy DSH kittens and also kittens with abnormal body condition.

## Materials and methods

### Study populations

Data were derived from BANFIELD® Pet Hospital (BANFIELD) clinical records and a research population. Data from client-owned DSH kittens were extracted from a copy of an Oracle 11g database (Oracle Corporation) of electronic medical records from BANFIELD, with all client names and addresses removed (henceforth referred to as the **client-owned pet data**). BANFIELD comprises a network of over 900 primary care veterinary hospitals located mainly in the USA. Bodyweight is routinely measured during consultations, whilst birth date and breed data are supplied by owners when the pet is first registered but not independently verified. The database covered the period between April 1994 and November 2016 although, due to growth in client numbers over this time, three quarters of the data were from 2004 onwards. **Research colony data** were derived from DSH cats housed at the Waltham Petcare Science Institute (WALTHAM) in the UK. These cats were maintained in environmentally-enriched housing and provided with a structured socialisation programme to meet their mental and physical needs. The type of research conducted at WALTHAM was primarily nutritional investigations, and all studies were conducted in accordance with the Animals (Scientific Procedures) Act 1986 and approved by the internal Animal Welfare and Ethical Review Body. All research colony data were stored in an electronic spreadsheet (Excel 2013, Microsoft Corp.).

### Data extraction and eligibility criteria

Both databases were searched for weight measurements from DSH cats under 2.5y age, calculated from measurement date and date of birth. In addition, kittens had to be sexually intact at the time of the weight measurement. Data from cats that were subsequently neutered were eligible for inclusion provided that they were sexually intact at the time the weight measurement was taken; after neutering, any further observations from that cat were excluded from the dataset. Observations for which information on age, sex or weight were unavailable were excluded as were those from cats whose records indicated a pregnancy before the age of 2.5y. Neutering dates were available for all neutered research colony cats and those that were castrated or spayed at a BANFIELD hospital; these dates were used to calculate neuter status at the time of each weight measurement. However, since neutering dates were not available for any client-owned DSH cats neutered outside of the BANFIELD network, such cats were excluded from the BANFIELD study population because their neuter status could not be confirmed.

Client-owned pet cat data were subject to additional eligibility criteria. First, only data from cats visiting for routine preventive healthcare or where the diagnosis was recorded as ‘healthy pet’ were included. Measurements were excluded from the dataset if the recorded bodyweight had been rolled over from a previous visit, or if the medical records indicated that the cat had been weighed whilst still in a pet carrier. Also excluded were all observations from cats where there was some doubt over the recorded sex (e.g., male cats that had apparently undergone ovariohysterectomy), or if the cat had received a diagnosis of a health condition associated with an altered pattern of growth before 4yrs of age (e.g., ‘diabetes mellitus’, ‘dwarfism’ or ‘hyperthyroidism’). Finally, only DSH kittens with at least one bodyweight recorded between the ages of 0.10 years (approximately 5 weeks) and 1.75 years (approximately 91 weeks) were included. This age range was chosen to ensure that the complete growth period was covered apart from the first 5 weeks, where data were too sparse to model reliably.

The client-owned DSH cats were assigned to three subsets, based upon the body condition scores recorded across the set of their visits. The **modelling subset** comprised data from DSH cats that had received a body condition rating of ‘normal’ or ‘ideal’ at one or more visits between the ages of 1.5 and 2.5 years (approximately 79 weeks to 130 weeks), taken as an indicator of having optimal body condition in young adulthood, and had never received an abnormal body condition rating (e.g., very thin, thin, heavy, overweight, markedly obese) at any point up to the age of 2.5 years (130 weeks). The **overweight subset** comprised data from DSH cats whose body condition was recorded as ‘heavy’, ‘overweight’ or ‘markedly obese’ between the ages of 1.5 and 2.5 years (approximately 79 weeks to 130 weeks) and had not received a body condition assessment of ‘normal’ during that age range. The **underweight subset** comprised data from DSH cats that had the equivalent definition for body conditions of ‘thin’ or ‘very thin’. The modelling subset was used to create the growth standards, whilst the overweight and underweight subsets were used for generating comparisons with the growth standards (described in detail below). For the research colony data, any cat receiving a body condition score (BCS) other than a 4 or 5 on a 9-point scale [15] at any age up to 2.5 years (approximately 130 weeks), were excluded.

### Generation and recording of clinical data

#### Client-owned DSH cats

For the client-owned pet data, the methods of recording signalment data (date of birth, breed and sex), bodyweight and clinical diagnoses in the BANFIELD clinical records have been previously described [5, 13]. Neuter status is also included in the record although, as described above, the date of neutering was only available when the procedure was carried out at a BANFIELD Hospital.

Body condition at BANFIELD was graded using a subjective 3-category body condition assessment (‘thin’, ‘normal’, or ‘heavy’) until 2010 after which a 5-category BCS scale was introduced (‘very thin’, ‘thin’, ‘ideal weight’, ‘overweight’, and ‘markedly obese’). However, since a substantial amount of the data extracted used the old 3-category assessment, the 5-category scale was mapped onto the 3-category scale for analysis (by merging ‘very thin’ and ’thin’, and ’overweight’ and ’markedly obese’), as previously described [13].

Until July 2013, BANFIELD veterinarians assessed body condition at their discretion, and if body condition was not assessed it remained at the default value of ’normal’ in BANFIELD clinical records. After this time, body condition assessment became a required field in the clinical software although, until March 2015, if a body condition assessment from a previous visit was available then this would roll forward to subsequent visits unless actively changed. After March 2015, the field remained mandatory and veterinarians were required to make an active selection at each visit. To ensure that only the data from an actual veterinary assessment were included, BCSs remaining at their default value (prior to July 2013) were converted to ‘unknown’ in the Oracle database copy of the client-owned pet data. However, for cats over 1.5 years (78 weeks) old, if a BCS had been recorded at a previous or subsequent consultation and was within ±5% of the weight recorded at a visit with unknown body condition, the BCS from that previous or subsequent visit (taking whichever was closest in age if there was more than one visit sufficiently close in weight) was used to replace the unknown one. A difference of ±5% was chosen because changes in bodyweight of >5% are typically required for changes in BCS to be seen in dogs [16], and this was assumed to be similar in cats.

#### Research colony cats

For the research colony data, body condition of cats was monitored regularly with corrective action taken where judged to be necessary by colony staff (e.g., reducing food intake when a cat had gained weight too quickly, based on experience of the carers when judging body condition). Body condition score was recorded on a 9-point scale [15].

### Data handling and statistical analysis

#### Sample size

A formal sample size calculation was not performed. Instead, the aim was to include as many cats as possible that met the eligibility criteria. However, the resulting modelling dataset was of comparable size to that used in a previous study to create growth charts for dogs [13].

#### Data cleaning

The distribution of the client-owned cat data was inspected during initial screening and weights that appeared to have been rounded to the nearest imperial pound mass (lb) were excluded. Other than this, the methodology for cleaning the datasets was as described previously [13], with some minor changes to account for the different age ranges and data characteristics of this study. In this respect, after excluding extreme outliers (i.e., bodyweight entries >3 times the median value for cats >9mo age), population outliers were identified by dividing the data into 50 equally-sized age groups fitting a loess-smoothed curve through the outlier limits for each group (defined as 175% of the upper and lower whiskers of a box-and-whisker plot) and excluding any points outside those lines as outliers. As previously described [13], additional data cleaning measures (‘within cat cleaning’) were then implemented for cats with repeat visit data to check the plausibility of recorded bodyweights against previous and subsequent measurements for the same individual. Finally, for the modelling data set, weights above 6.5 kg were filtered out as being unlikely for a DSH cat of normal BCS, except where these could be verified against other weights from that individual as described above.

#### Creation of growth centile curves

Growth centile curves were constructed using Generalised Additive Models for Location, Shape and Scale (GAMLSS) [17], which is the same model class that the WHO Multicentre Growth Reference Study Group used to construct the WHO Child Growth Standards [11] and which the authors previously used to create growth standards for dogs [13]. GAMLSS is a semi-parametric modelling technique, whereby aspects of the underlying distribution (central tendency, spread, skewness and kurtosis) are estimated as smooth functions of the predictor variable(s). The specific type of GAMLSS model used was the BCCG (Box-Cox Cole-Green) model. This was fitted to the modelling subset with the dependent variable being bodyweight and the independent variable being age (raised to the power of 0.1 to improve the model fit as described previously [5, 13]). Separate models were built for male and female DSH cats. Further details of the modelling process were as described previously [13].

The models were displayed graphically as centile curves covering the age range 8 weeks to 78 weeks and showing centiles 0.4%, 2%, 9%, 25%, 50%, 75%, 91%, 98% and 99.6%. These centiles were the same as those used in the UK-WHO growth charts [18] and equally spaced on the z-score (standard deviation) scale, which is advantageous from an arithmetic point of view.

### Comparison of other growth data to growth centile curves

The growth patterns depicted by the growth centiles curves were compared with the growth patterns of cats contributing to the research colony data (assessing ‘healthy growth’) and both the overweight and underweight subsets of the client-owned data (assessing ‘potentially-abnormal growth’).

#### Population-level analysis

For each dataset, bodyweights were modelled by age (raised to the power of 0.1) using BCCG GAMLSS models separately for male and female, as described previously [5, 13]. The predicted median (50%) and inter-quartile weights (25% and 50%) were extracted at intervals of 0.05y between the ages of 0.15y and 1.5y. These were converted to centiles according to the sex-appropriate growth standard. This converts centile curves to horizontal straight lines, so that abnormal growth appears as a rising or falling line [5]. These centiles were then plotted against age, separately for each sex, to illustrate how the median and interquartile growth in each data set compared to the growth standard.

#### Individual-level analysis

Observations from DSH kittens with at least 3 visits over at least a 6-month period between the ages of 8 weeks and 78 weeks were extracted from each data set. These were converted to the z-score scale according to the sex-appropriate growth standard. The differences between the z-score at the initial visit and the greatest and least z-scores attained by that kitten were calculated and divided by 0.67 to convert to the number of standard centile lines crossed in an upwards / downwards direction during that individual’s growth. This divisor was used because the standard centiles, when converted to z-scores, were equidistant, with this being the separation between successive centiles. The number of centile crossings in each data set were compared graphically, using density plots, and statistically, by fitting a GAMLSS zero-adjusted gamma model and comparing the coefficients, using Tukey post-hoc comparisons and simulation.

#### Software

All analyses were performed with an open-access online statistical software environment (R, version 3.6.1 [19]), using the R package gamlss [17] for the GAMLSS modelling and the multcomp package [20] for post-hoc comparisons. Graphics were produced using ggplot2 [21].

## Results

### Data handling / cleaning and construction of growth curves

As these growth standards were calculated from a large observational dataset, a sequence of data cleaning steps was needed, as previously described. Table 1 shows the number of rows of data and individual cats in the datasets at each stage in the data cleaning process from initial data extraction to final datasets.

**Table 1:** Details of data sets at each stage of data cleaning.

Data Cleaning Stage	Data Set							
	Modelling Data		Research Colony Data		Overweight Subset		Underweight Subset	
	Male	Female	Male	Female	Male	Female	Male	Female
Data Extraction (RAW DATA)	10,873 rows 4,477 cats	14,388 rows 5,800 cats	24,557 rows 566 cats	26,474 rows 557 cats	30,883 rows 11,401 cats	23,659 rows 8,564 cats	617 rows 292 cats	861 rows 434 cats
Removal of Whole lb Weights	9,129 rows 4,039 cats	12,142 rows 5,246 cats	24,557 rows 566 cats	26,474 rows 557 cats	25,694 rows 10,436 cats	19,637 rows 7,845 cats	455 rows 232 cats	643 rows 337 cats
Removal of Extreme Outliers	9,128 rows 4,039 cats	12,139 rows 5,245 cats	24,556 rows 566 cats	26,474 rows 557 cats	25,692 rows 10,436 cats	19,637 rows 7,845 cats	455 rows 232 cats	641 rows 335 cats
Population Level Cleaning	8,900 rows 3,961 cats	11,606 rows 5,126 cats	23,811 rows 564 cats	25,684 rows 557 cats	24,412 rows 10,210 cats	18,148 rows 7,685 cats	441 rows 225 cats	618 rows 328 cats
Removal of Duplicate Observations	8,812 rows 3,950 cats	11,495 rows 5,148 cats	23,808 rows 564 cats	25,684 rows 557 cats	24,390 rows 10,210 cats	18,122 rows 7,685 cats	440 rows 225 cats	614 rows 328 cats
Within-Cat Cleaning	6,274 rows 3,116 cats	7,983 rows 3,997 cats	23,645 rows 563 cats	25,484 rows 545 cats	17,199 rows 7,943 cats	12,640 rows 6,060 cats	299 rows 175 cats	417 rows 255 cats
Exclude Singleton Weights of >6.5kg	6,254 rows 3,103 cats	7,961 rows 3,987 cats	N/A	N/A	N/A	N/A	N/A	N/A
FINAL DATA	6,254 rows 3,103 cats	7,961 rows 3,987 cats	23,645 rows 563 cats	25,484 rows 545 cats	17,199 rows 7,943 cats	12,640 rows 6,060 cats	299 rows 175 cats	417 rows 255 cats

**Fig 1:**
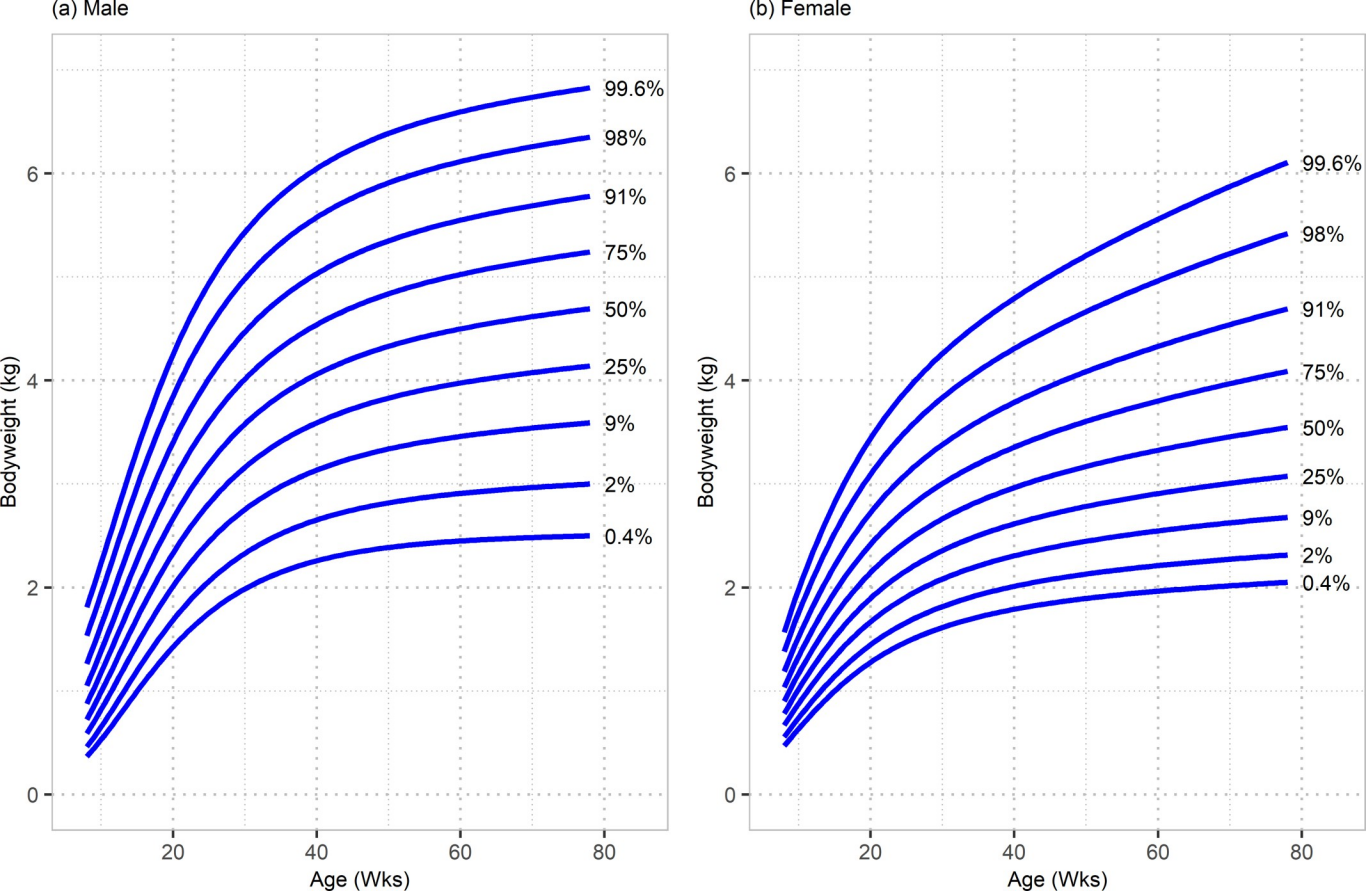
Growth standard chart for male and female DSH cats. Plots are split by sex: (a) male and (b) female. The x-axis depicts age in weeks, whereas the y-axis depicts bodyweight in kilograms.

Separate sets of growth centile curves were successfully constructed for male and female DSH kittens using the modelling data set (Fig 1).

### Population-level analysis

#### Investigating growth trajectories in healthy cats from a research colony

**Fig 2:**
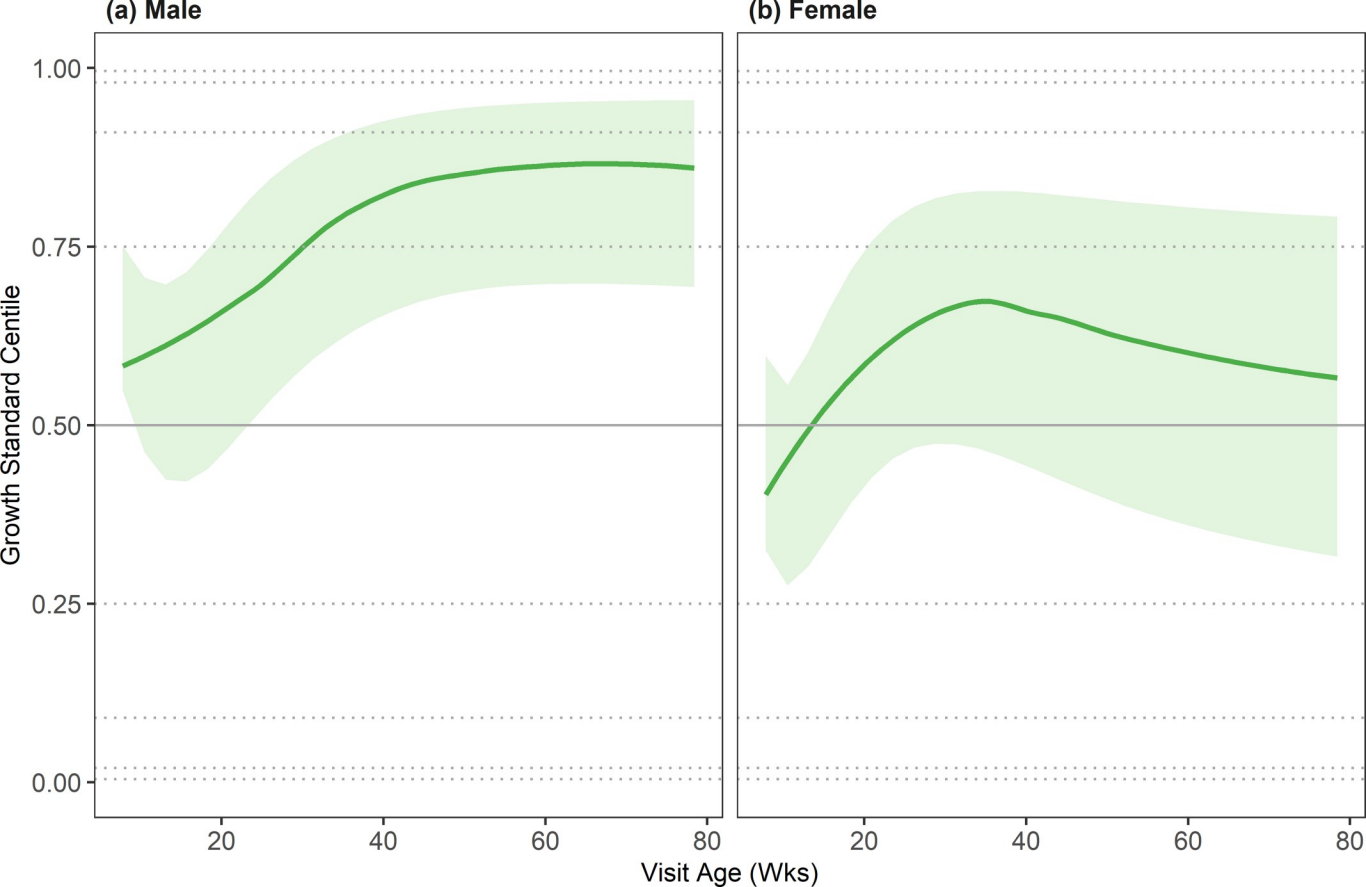
Median and interquartile weight by age (on the centile scale) for DSH kittens in the research colony data set. Plots are split by sex: (a) male and (b) female. On each graph, the median is depicted by the solid green line, the interquartile range by the shaded region, and the standard centiles (0.4%, 2%, 9%, 25%, 50%, 75%, 91%, 98% and 99.6%), as calculated from the growth standard population, are shown by the grey horizontal lines (dotted, apart from 50%, which is shown as solid).

To assess whether the growth standards reflected growth patterns of healthy DSH kittens, they were compared with average growth trajectories from a research colony. Fig 2 shows the predicted median growth trajectories and interquartile ranges for male and female research colony kittens superimposed on the growth standard centiles. These are shown on the centile scale, which means that a trajectory following the growth standard is plotted as a horizonal line; an alternative presentation, with the median growth trajectory shown on the kg scale, is shown in S1 Fig. For male DSH kittens (Fig 2A), the median trajectory started a little above the median centile line (57^th^ centile) and rose, crossing the 75^th^ centile at about 7 months of age, before levelling out 75^th^ and 91^st^ centile lines at about 10 months of age. For female DSH kittens (Fig 2B), the median trajectory started just below the 41^st^ centile line, rising to a maximum of the 67^th^ centile just after 6 months of age before decreasing again, but always lying between the 25^th^ and 75^th^ centile lines and between the median and 75^th^ centile lines for most of the age range. Therefore, the male research colony cats ended up, on average, tracking a slightly higher centile after 40 weeks of age than the one that they started on, whereas the female cats showed much less deviation with the average trajectory staying close to the median centile. Overall, therefore, the growth of DSH kittens tracked the growth centile curves relatively well.

#### Investigating predicted growth trajectories in cats with abnormal body condition

**Fig 3:**
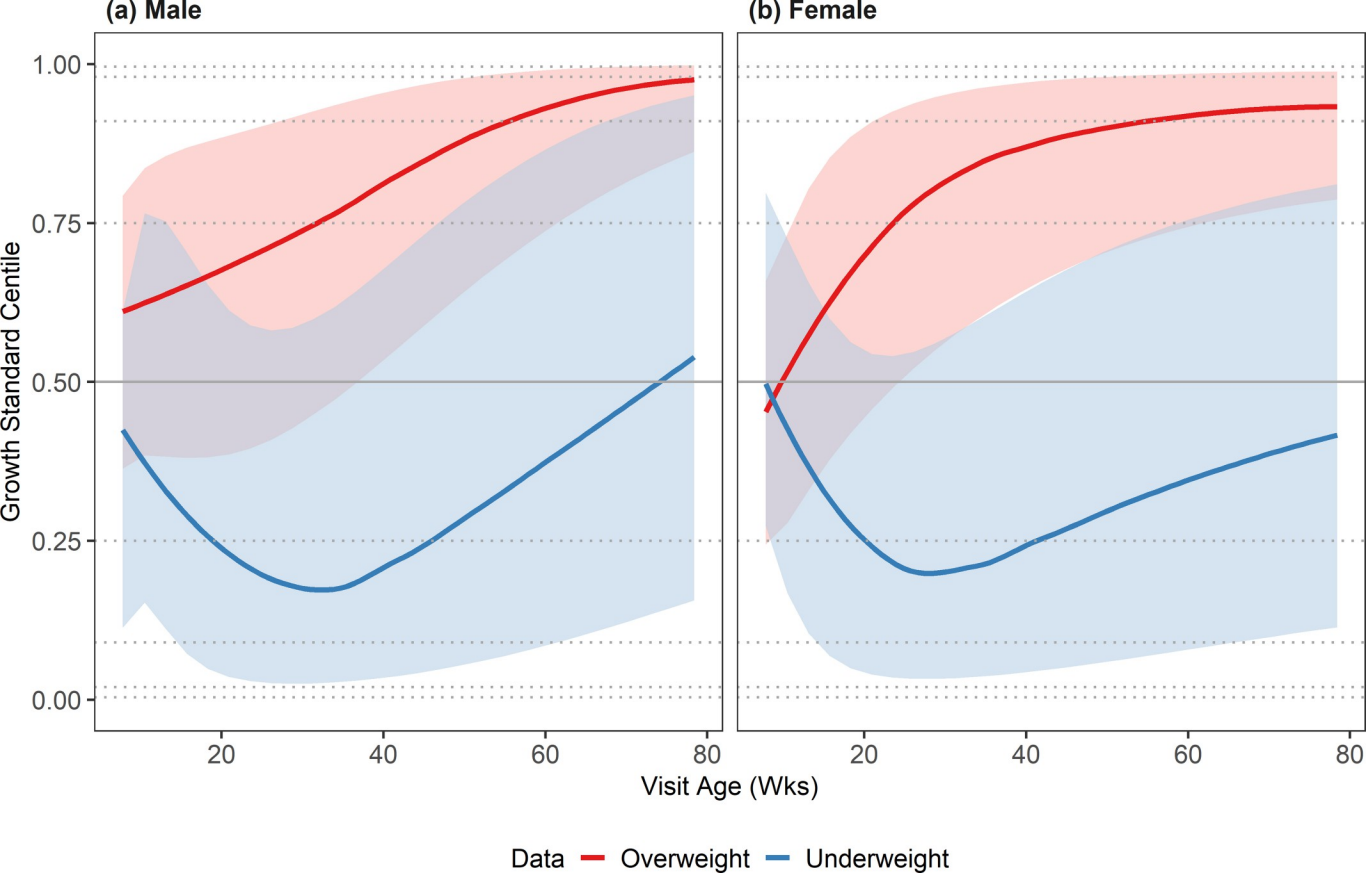
Median and interquartile weight by age (on the centile scale) for DSH kittens in the underweight subset (blue) and overweight subset (red). Plots are split by sex: (a) male and (b) female. On each graph, the median is depicted by the solid blue/red line, the interquartile range by the shaded region, and the standard centiles (0.4%, 2%, 9%, 25%, 50%, 75%, 91%, 98% and 99.6%), as calculated from the growth standard population, are shown by the grey horizontal lines (dotted, apart from 50%, which is shown as solid). Some centile crossing is observed in both groups up to 20-30wks but the overweight kittens continue to cross centiles throughout the growth period.

To assess if the standards differed from the growth trajectories from DSH kittens with non-ideal development, the standards were compared to average trajectories from DSH kittens with known body condition score issues. Fig 3 shows the predicted median growth trajectories and interquartile ranges (IQR) for male and female DSH kittens of the underweight and overweight subsets, superimposed on the growth standard centiles. As with the plots for the healthy research colony kittens, these are shown on the centile scale meaning that a trajectory following the growth standard would be plotted as a horizonal line; an alternative presentation, with the median growth trajectory shown on the kg scale, is shown in S2 Fig. For the underweight subset, growth trajectories of both sexes were similar, with a marked initial dip (indicating slower growth than the growth standards) reaching a minimum point of the 17^th^ and 19^th^ centiles for males and females, respectively, at about 7 months of age. After this age, rate of growth was faster than that depicted in the growth standards suggesting a degree of catch-up growth. In the overweight subset, male kittens (Fig 2A) were heavier on average at the start of monitoring (starting at the 59th centile) and growth rate was faster than that depicted in the growth standards throughout the growth period, reaching the 75^th^, 91^st^ and 98^th^ centiles by 34, 55 and 79 weeks of age, respectively. The median starting weight of female kittens (Fig 2B) was slightly less than that predicted by the standards (44^th^ centile), but the subsequent rate of growth was faster than that expected according to the growth standards (and indeed faster than that seen in the male kittens), crossing the 75^th^ and 91^st^ centiles by 24 and 53 weeks of age, respectively.

### Individual-level analysis

Following the comparisons with average trajectories from kittens with healthy and unhealthy development, the next step was to enumerate what amount of departure from the standards could be considered normal in healthy kittens, and how this differed from that seen in kittens with body condition issues.

#### Investigating individual growth in healthy cats from a research colony

**Fig 4:**
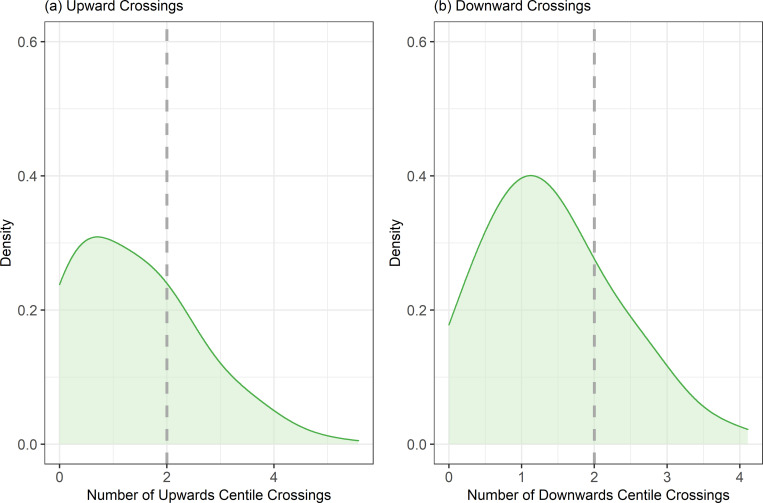
Density distribution of the number of standard centile lines crossed in an (a) upwards and (b) downwards direction for cats in the Research Colony data set. Plots only include kittens that had at least 3 weight measurements over 6 months in the age range 8 to 78 weeks old. The vertical dotted line indicates 2 centile crossings.

After the additional data filtering to extract cats with at least 3 visits over 6 months between 8 and 78 weeks of age, there were 33,274 bodyweight measurements (from 507 cats) for the research colony data set. For an individual growth trajectory, crossings were calculated as the maximum deviation (expressed as number of standard centile lines crossed and expressed fractionally if necessary) in the stated direction from the initial centile line. Fig 4 shows the density distributions of the number of centile crossings upwards and downwards in the research colony dataset across both sexes. A total of 142 (28%) and 119 (23%) kittens, crossed at least 2 standard centile lines in an upwards and downwards direction, respectively.

#### Investigating individual growth in DSH kittens with abnormal body condition

**Fig 5:**
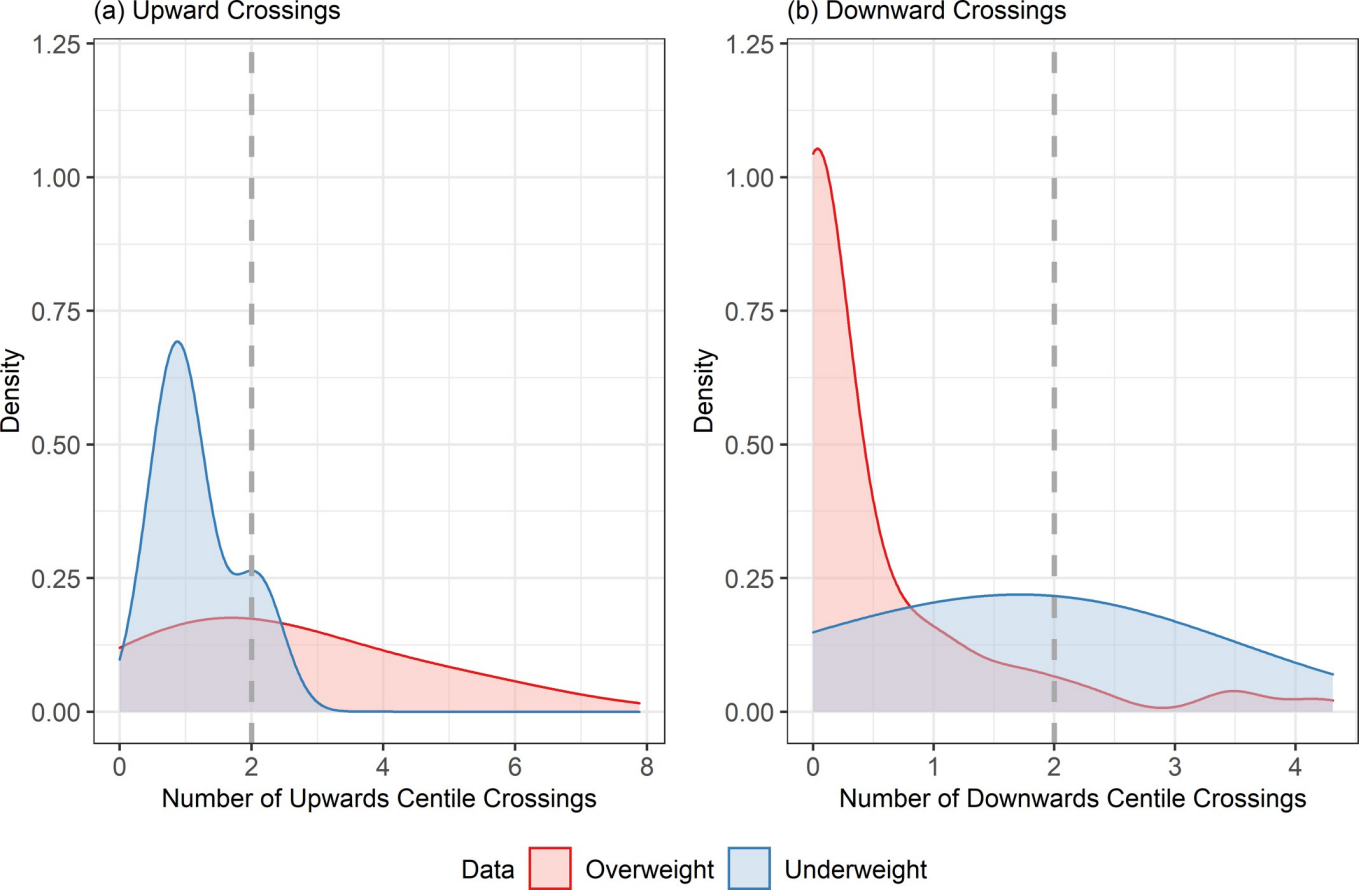
Density distribution of the number of standard centile lines crossed in an (a) upwards and (b) downwards direction for DSH kittens. Plots only include those with at least 3 weight measurements over 6 months in the age range 8 to 78 weeks old in the underweight subset (blue) and overweight subset (red). The vertical dotted line indicates 2 centile crossings.

After the additional data filtering, there were 513 bodyweight measurements (from 122 cats) for the overweight subset and 23 bodyweight measurements (from 4 cats) for the underweight subset, and Fig 5 shows the density distributions of the number of upwards and downwards centile crossings across both sexes. About half of the overweight kittens (63, 52%,) crossed at least 2 standard centile lines in an upwards direction, compared with 1 kitten (25%) in the underweight subset. Conversely, 2 of the underweight kittens (50%) crossed at least 2 standard centile lines in a downwards direction, compared with 8 kittens (7%) in the overweight subset. Although the small number of underweight kittens with repeated visits complicates the interpretation, this suggested that crossing 2 or more standard centile lines upwards or downwards is more likely in cats with abnormal body condition.

#### Statistical comparisons of the pattern of growth amongst datasets

In order to quantify how the patterns of crossings differed between the experimental groups, zero-adjusted gamma models were fitted. Table 2 shows the fitted values of the coefficients for these models together with 95% confidence intervals (95%-CI) and Tukey post-hoc comparison groups. For the upward crossing model, Mu (the location measure for the number of centile crossings) was greater for the overweight subset than for either of the other groups (*P*<0.001, for both) indicating that, amongst the cats that had upward crossings, those in the overweight group crossed more standard centile lines than cats in the other groups. For the downward crossings model, Mu was greater for the underweight group than for the overweight group (*P* = 0.011), although there was no difference between the underweight and research colony data (*P* = 0.137). This indicated that, amongst cats with downward crossings, those in the underweight group crossed more standard centile lines downwards than those in the overweight group.

**Table 2:** Comparison of upward and downward crossings in the three comparison datasets.

Model Parameter	Link Function	Data Set	Downward Crossings		Upward Crossings	
			Estimate (95% CI)	Tukey Group^2^	Estimate (95% CI)	Tukey Group^2^
Mu (mean for non-zero values)	Log	Research Colony	0.393 (0.323, 0.464)	b	0.447 (0.352, 0.541)	ab
		Overweight Subset	0.0624 (-0.303, 0.428)	a	0.972 (0.746, 1.20)	a
		Underweight Subset	0.723 (0.298, 1.15)	b	0.155 (-0.382, 0.691)	b
Sigma (coefficient of variation for non-zero values)	Log	Research Colony	-0.435 (-0.507, 0.363)	a	-0.169 (-0.241, -0.0972)	b
		Overweight Subset	0.0808 (-0.143, 0.304)	a	0.200 (-0.376, -0.0248)	a
		Underweight Subset	-1.20 (-2.17, -0.240)	a	-0.828 (-1.65, -0.00601)	b
Nu (probability of a zero value)	Logit	Research Colony	-2.88 (-3.35, -2.41)	a	-2.07 (-2.40, -1.73)	b
		Overweight Subset	0.231 (-0.559, 1.020)	a	-2.66 (-3.65, -1.67)	a
		Underweight Subset	-1.10 (-3.91, 1.71)	a	-11.2 (-206, 184)	ab

^1^ Values of coefficients (with 95%-CI) for the zero-adjusted gamma model fitted to the number of upward and downward crossings in each of the three comparison datasets, together with p-values. The three parameters of the zero-adjusted model correspond to the probability of obtaining a zero value (Nu), the mean of the distribution for non-zero values (Mu) and the coefficient of variation for non-zero values (Sigma).

^2^ Tukey groups indicate whether differences exist between groups for a parameter within a model; groups with different letters are significantly different at the 5% significance level.

Simulation (using 10,000 values) from these models indicated that the overall mean number (95%-CI) of upward crossings was 1.39 (1.23, 1.56) for the research colony data, 2.47 (1.84, 3.21) for the overweight subset and 0.67 (0, 2.10) for the underweight subset, whilst the mean number of downward crossings was 1.40 (1.28, 1.53) for the research colony data, 0.48 (0.22, 0.86) for the overweight subset and 1.47 (0.19, 2.87) for the underweight subset. There were more upwards crossings in the overweight group than either of the other two groups (mean [95%-CI] difference between the overweight and underweight groups = 1.80 [0.14, 3.14] and between the overweight and research colony groups = 1.08 [0.43, 1.85]). However, there was no difference between the underweight and research colony data (mean -0.72 [95%-CI: -1.53, 0.73]). For the downwards crossings, there were no differences between any of the groups at the 5% significance level. Overall, these results suggest that the research colony data were quite balanced between upward and downward crossings, whilst the overweight and underweight subsets were more biased towards upward and downward crossings, respectively.

## Discussion

In the current study, we first utilised data from a large population of healthy mixed-breed pet DSH cats to create growth charts for male and female kittens, and then compared growth depicted by the curves of the standard with growth patterns of separate groups of kittens, including healthy individuals and those with abnormal body condition. These charts could underpin a clinical tool to assist veterinary professionals in monitoring growth of kittens during their early life. Such a tool could be used to promote healthy growth and identify those with abnormal patterns of growth. As previously discussed, kittens that grow more quickly are at risk from developing obesity later in life [7]. Identifying such kittens could enable preventive measures to be implemented before obesity has had a chance to develop. Therefore, although the tool is directly relevant for kittens, its impact stretches into adulthood.

The GAMLSS modelling used in the current study was equivalent to those used to create the WHO Child Growth Standards [10–12], which monitor growth of children globally. The latter are a ‘growth standard’, rather than a ‘growth reference’. Whereas a growth reference describes growth in a defined population, without making any inference to health, a growth standard represents an ideal, by describing growth of ‘healthy’ infants [22]. For the WHO Child Growth Standards, the infants studied were exclusively or predominantly breastfed, raised in favourable socioeconomic conditions by non-smoking mothers and were not subjected to problems likely to constrain growth [11]. Therefore, such a reference depicts optimal growth in infants achieving their full genetic potential. In the current study, only healthy cats were selected to participate–those attending a routine preventative healthcare visit or those where the diagnosis was recorded as ‘healthy pet’. Furthermore, all participating cats remained in ideal body condition throughout the first 3 years (approximately 156 weeks) of life. Therefore, as with the WHO Child Growth Standards, the charts created for kittens in the current study would also be a growth standard.

The growth trajectories depicted by the curves of the newly-created growth reference standards for DSH kittens, were then compared with growth patterns in other healthy kittens. For this, a group of cats from a research colony were used. On individual analysis, although centile crossing was relatively common, it could occur in either direction with 28% and 23% of the kittens crossing 2 standard centile lines in an upwards and downwards direction, respectively. However, in female kittens, the maximum point reached was the 86th centile, suggesting a median growth pattern similar to the population used to create the standard staying within one line of the 50th centile. Conversely, the male kittens from the research colony were somewhat bigger than the standard, with both a heavier median starting weight and initial rate of growth, which subsequently settled down to follow a standard (albeit higher) centile curve from 10 months onwards. Despite these differences, growth remained within 2 lines of the 50th centile. The reason for the different growth pattern in male kittens is not known but might be due to differences in stature of the male cats in this UK research colony, compared with the stature of the cats used for the modelling dataset which was from a veterinary hospital network in the USA. Although this network is large and covers a wide geographic region, patients attending these veterinary practices might differ from those of other types of practice (e.g., independent veterinary clinics) or practices from other countries. In addition to genetic differences, there might be differences in husbandry in other regions. Historically in this research colony, larger male kittens tended to be selected for the breeding stock, and these were included in the population studied. Not uncommonly, minor differences can be seen in patterns of healthy growth between populations; for example, compared with the WHO growth standards; for example, relative to the WHO growth standards, British children tend to be a heavier weight at the time of birth but, thereafter, grow slightly more slowly [23]. Although the use of cats from a research colony has the advantage of yielding readily available, accurate and mostly complete historical data, one disadvantage is that cats might not be representative of pet cats both genetically and because of the environment in which they are maintained. Therefore, ideally, further studies are needed, to assess growth patterns in different groups of healthy pet kittens (from different geographic regions, for example), so that the validity of these standards can be confirmed within the wider population. Such studies are commonplace when validating growth standards, for example, the work underpinning the WHO growth standards [10–12, 23].

Growth patterns of pet cats that a veterinarian had classified as overweight and underweight were also compared with these new growth standards. Cats in overweight body condition tended to have crossed centile lines upwards suggesting that they had grown at a faster rate than the standard population. The median weight of the overweight subset had crossed the 91^st^ centile before reaching skeletal maturity, with about half of this group crossing two or more centiles in an upwards direction. In contrast, there was some downwards crossing of centiles in the underweight subset, although this was less marked than upwards crossings in the overweight subset. The trajectory seen in this this subset was notable with a marked initial dip, compared with the standards, and subsequent catch up. Although the reason for this pattern is unclear, it is possible that the subsequent catch up reflects corrective action on the part of the owner and veterinary professional after a kitten was found to be underweight. An alternative possibility is that, because the underweight subset was much smaller than the overweight subset, the statistical analyses were relatively underpowered and observed relationships subject to statistical ‘noise’. Therefore, further work examining growth patterns in cats that grow too slowly is needed, perhaps, examining patterns in those with disorders known to affect growth, as has been undertaken for children [24] and dogs [5].

Notably, a difference in growth pattern could be identified relatively early during the growth period, with the 25^th^ and 75^th^ centiles being crossed by 19 and 24–34 weeks, in the underweight and overweight subsets, respectively. These findings suggest that the growth standards created could form the basis for use as a clinical tool for monitoring healthy growth in DSH kittens. In this respect, if a kitten is found to cross centiles, its nutrition and husbandry can be reviewed and diagnostic investigations could be undertaken if necessary. If a kitten is growing more quickly than expected, its food intake could be reduced. Conversely, where a kitten is growing more slowly than expected, diagnostic investigations should be undertaken to determine the underlying cause. Depending upon the cause, it might also be necessary either to increase intake or change the type of food. Although uncommon, some upwards and downwards centile crossing was seen in cats of the underweight and overweight subsets, respectively. One possible reason for this apparent inconsistency of centile crossing in kittenhood with adult body condition is that some cats only became underweight or overweight after the end of the growth period. Alternatively, a potentially more likely explanation for this paradoxical crossing might be the result of attempts to correct abnormal body condition after first being identified. Similar results are reported with human growth standards. For example, crossing centiles upwards is associated with greater odds of a child developing obesity, but this is not inevitable [12]. Therefore, such growth standards are best used as an aid to support decision making by veterinary professionals about the need for changes to nutrition and husbandry.

When judging the impact of this study, several limitations must be considered, some of which are similar to those discussed about the work developing puppy growth standards [5]. These include that the data were collected over many years, possible inaccuracies in data entry and uncertainties around some of the data gathered (e.g., example pet dates of birth as supplied by owners). Prior to data analysis, a significant amount of data cleaning was needed, on account of issues such as peaks in the weight data corresponding to rounding by veterinarians. Ideally other growth measurements such as length and body circumference would have been gathered, but these were not available in the retrospective data used. Such length measurements are used for monitoring the growth of children but are not routinely collected by veterinarians. Prospective studies would be required to examine such changes during the growth period.

A second limitation is that we chose only to analyse data from sexually-intact cats, albeit that data were included from many before they were neutered. Given the known association between neutering and obesity in cats [25–28], it is possible that growth patterns are affected by either castration or ovariohysterectomy. That said, only a limited impact of neutering on the growth of healthy puppies was observed in a recent study that used similar growth standards [18], where early neutering resulted in a modest increase in growth rate, whilst neutering at a later stage was associated with a modest decrease in growth rate [5]; however, deviations were minimal and remained within a single centile unit. Nevertheless, before the current standards can be used to monitor growth patterns in DSH kittens, further work is required to assess the potential impact of neutering.

A third study limitation was the fact that, given our reliance on electronic health records, we had no information about the kittens from birth to weaning, such as whether they were underweight at birth and how they developed during the early life period. This also we lacked information about the maternal environment, which is known to affect the growth and development in other species such as sheep [29]. Further work would be required to examine such influences in the development and growth in cats.

A fourth study limitation was the fact that the comparison data from healthy cats were relatively limited and were derived from cats maintained in a research colony. Such cats might not be representative of client-owned cats as a result of, for example, the inclusion of cats chosen for (future) breeding. In contrast, the datasets used to assess abnormal body condition were more representative because they were derived from cats attending the same veterinary hospital network as those used in the modelling dataset. These datasets were larger although there were still some issues, notably the very limited number of cats assigned to the underweight dataset. A final consideration was the fact that the growth standards have been created from DSH cats. Given that these represent the majority of the feline pet population, the standards are likely to be applicable to many pet cats seen by veterinarians. However, further work would be required to determine how patterns of growth differ in pedigree breeds and domestic medium/long hair cats, and whether bespoke standards might be required.

## Conclusion

A series of evidence-based growth standards, based on bodyweight, have been developed for male and female DSH kittens. Patterns of growth in cats with abnormal body condition have also been compared with the growth curves depicted by the standards. Although not perfect, crossing centiles in an upwards and downwards direction is associated with cats reported to be overweight or underweight by early adulthood, respectively. Prospective studies are now required to determine whether use of these growth standards by veterinarians in clinical practice will improve the health and wellbeing of cats under their care and decrease the risk of cats entering their adult years in overweight condition.

## Supporting information

S1 Fig Median and interquartile weight by age for kittens in the research colony data set. Plots are split by sex: (a) male and (b) female. On each graph, the median is depicted by the solid green line, the interquartile range by the shaded region, and the growth standard centiles (0.4%, 2%, 9%, 25%, 50%, 75%, 91%, 98% and 99.6%) are shown by the grey lines (dotted, apart from 50%, which is shown as solid).
(TIF)

S2 Fig Median and interquartile weight by age for kittens in the underweight subset (blue) and overweight subset (red). Plots are split by sex: (a) male and (b) female. On each graph, the median is depicted by the solid blue/red lines, the interquartile range by the shaded region, and the growth standard centiles (0.4%, 2%, 9%, 25%, 50%, 75%, 91%, 98% and 99.6%) are shown by the grey lines (dotted, apart from 50%, which is shown as solid).
(TIF)
