# Supplementary figures and images for: Growth standard charts for monitoring bodyweight in intact domestic shorthair kittens from the USA
Source: PLoS One. 2022 Nov 21;17(11):e0277531. doi: 10.1371/journal.pone.0277531 (PMC9678321; doi:10.1371/journal.pone.0277531)

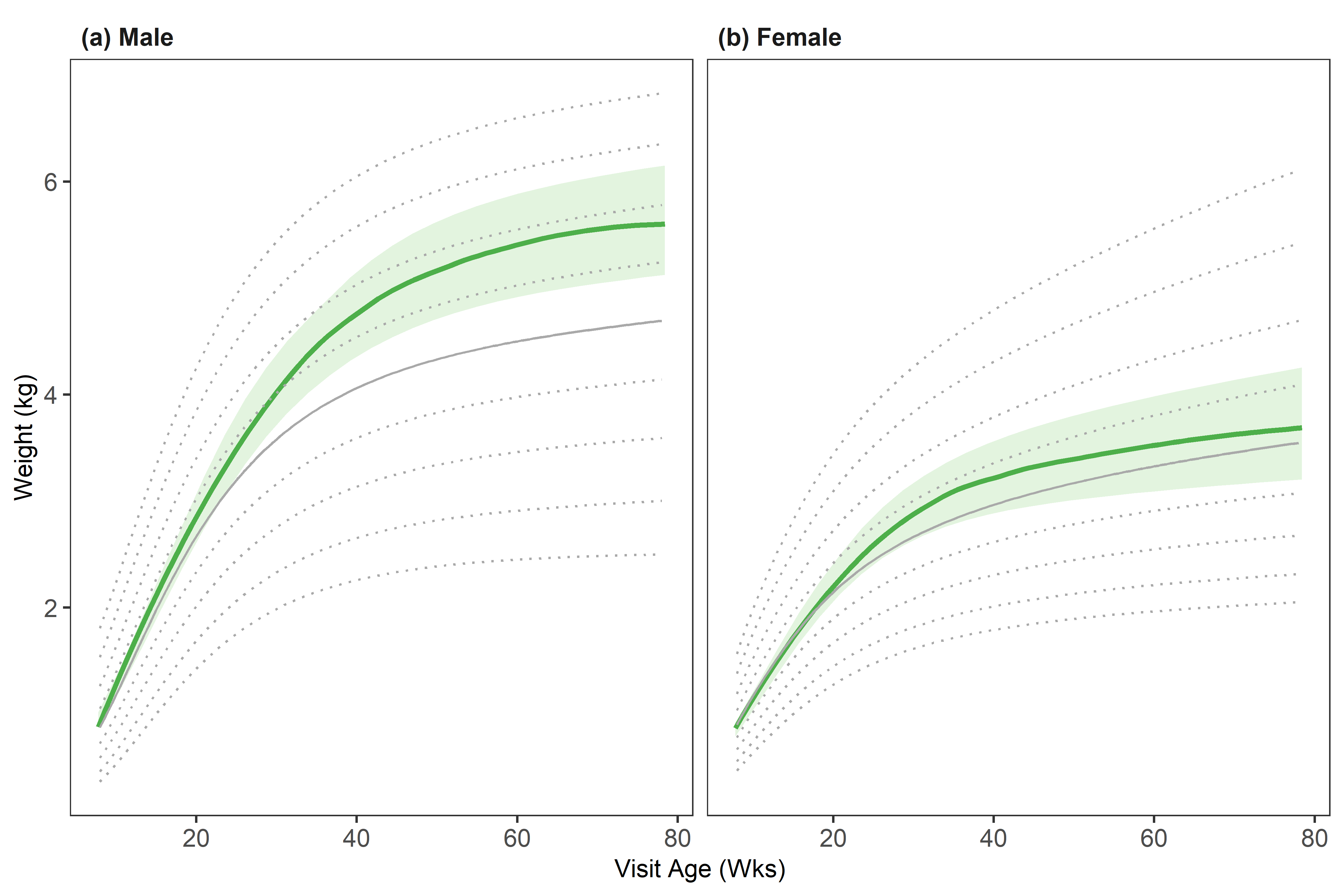

Supplement: S1 Fig — Plots are split by sex: (a) male and (b) female. On each graph, the median is depicted by the solid green line, the interquartile range by the shaded region, and the growth standard centiles (0.4%, 2%, 9%, 25%, 50%, 75%, 91%, 98% and 99.6%) are shown by the grey lines (dotted, apart from 50%, which is shown as solid). (TIF) [file pone.0277531.s001.tif]

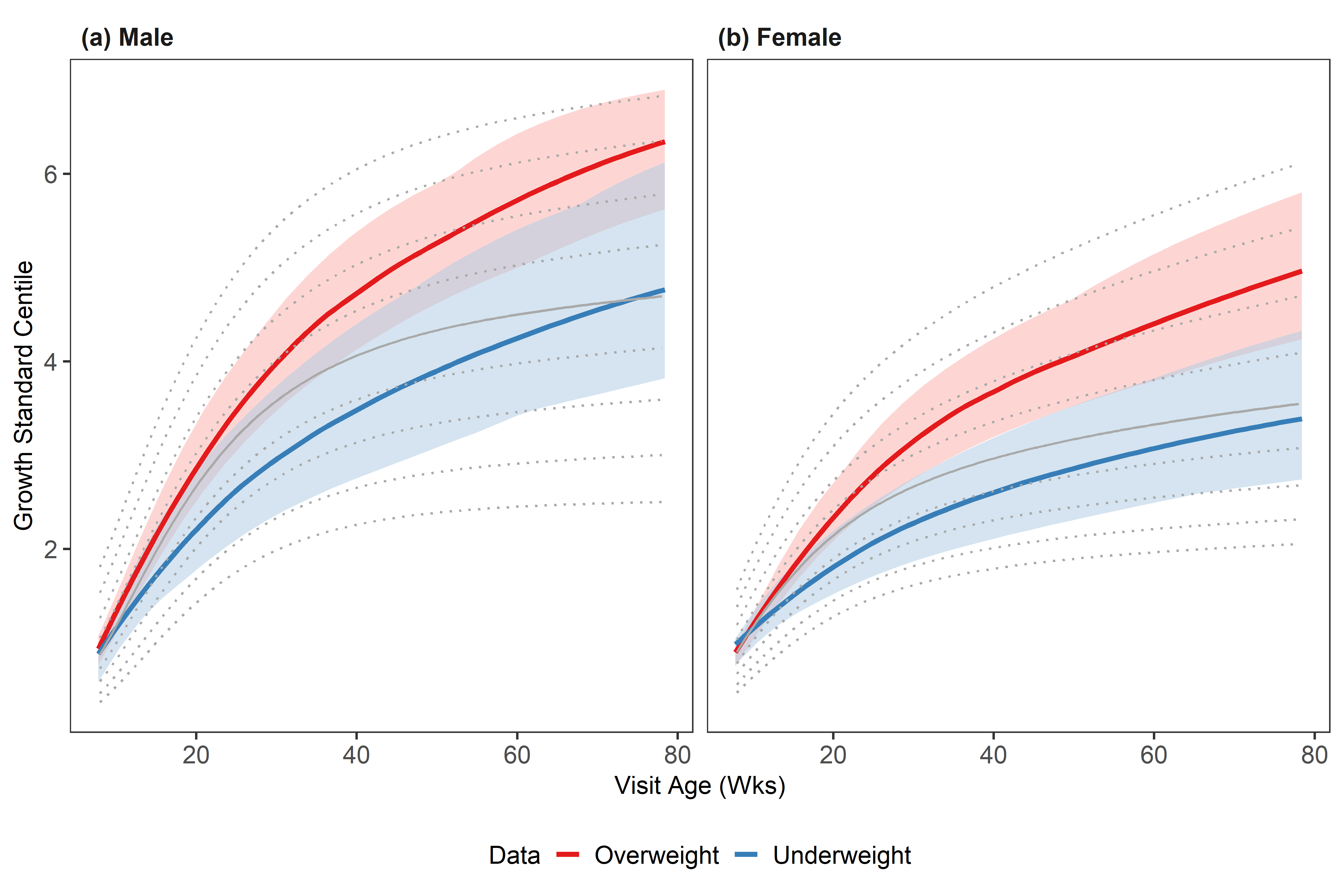

Supplement: S2 Fig — Plots are split by sex: (a) male and (b) female. On each graph, the median is depicted by the solid blue/red lines, the interquartile range by the shaded region, and the growth standard centiles (0.4%, 2%, 9%, 25%, 50%, 75%, 91%, 98% and 99.6%) are shown by the grey lines (dotted, apart from 50%, which is shown as solid). (TIF) [file pone.0277531.s002.tif]
